# “When the virus decompensated the neurosis.” About a case

**DOI:** 10.1192/j.eurpsy.2022.1337

**Published:** 2022-09-01

**Authors:** M. Valverde Barea, A. España Osuna, P. Vargas Melero, M. Solis

**Affiliations:** 1 Universitary Hospital of Jaén, Psychiatric, Jaén, Spain; 2 Universitary Hospital of Jaén, Psychiatric, Jaen, Spain

**Keywords:** Covid-19, Depression, Hypochondriacal disorder, psychotic symptoms

## Abstract

**Introduction:**

The COVID-19 pandemic and social and mobility restriction measures have had a negative impact on the mental health of the population.

**Objectives:**

The objective is to demonstrate the impact of the pandemic on mental disorders.

**Methods:**

64-year-old man who is taken to the emergency room after a suicide attempt, by hanging with a belt out of concern and measuring the contagion of the COVID-19 virus in the context of long-standing delirious ideas of contamination and hypochondriacal neurosis. Adaptive disorder in relation to previous divorce. Psychopathologically, the patient is anxious and restless, conscious, inattentive and poorly oriented in space and time. Accelerated language with monothematic discourse about the possibility of contagion that has caused isolation behavior to the point of shredding organic waste and throwing it down the toilet so as not to have to go out to throw it out for fear of contagion. Faced with a neighbor’s wake-up call due to a blocked pipe, he suffers a crisis of guilt and anxiety and attempts to commit suicide. COVID-19 PCR=negative. Beck’s Depression Inventory 24=moderate depression. IPDE accentuated obsessive and avoidant personality traits.

**Results:**

Diagnosis: Moderate depressive episode with psychotic symptoms. Hypochondriacal disorder. Ananchastic personality disorder. Treatment: Paliperidone 3mg/24h. Sertraline 100mg/24h

**Conclusions:**

In obsessive personalities and hypochondriacal neuroses, the COVID-19 pandemic has posed an increased risk of decompensation for affective disorders and even suicide attempts. Isolation, lack of treatment and prior monitoring, as well as the difficulty of identifying vital stressors, must be taken into account if an early intervention is to be carried out.

**Disclosure:**

No significant relationships.

